# NDRG2 plays a pivotal role in ATLL leukaemogenesis by regulating the PTEN-mediated PI3K/AKT signalling pathway

**DOI:** 10.1186/1742-4690-11-S1-P87

**Published:** 2014-01-07

**Authors:** Shingo Nakahata, Tomonaga Ichikawa, Yusuke Saito, Yasuhito Arai, Tomohiko Taki, Masafumi Taniwaki, Kazuhiro Morishita

**Affiliations:** 1 Division of Tumor and Cellular Biochemistry, Department of Medical Sciences, University of Miyazaki, Kiyotake, Miyazaki, Japan; 2 Division of Cancer Genomics, National Cancer Center Research Institute, Chuo-ku, Tokyo, Japan; 3 Department of Molecular Diagnostics and Therapeutics, Kyoto Prefectural University of Medicine, Kamigyo-ku, Kyoto, Japan; 4 Department of Molecular Hematology and Oncology, Kyoto Prefectural University of Medicine, Kamigyo-ku, Kyoto, Japan

## 

Adult T-cell leukemia/lymphoma (ATLL) is a rare and aggressive T-cell leukemia/lymphoma that is etiologically linked to infection by human T-cell lymphotropic virus type 1 (HTLV-1). To find candidate causative genes for ATLL development, we used an integrative genomic analysis of ATLL and identified N-myc downstream-regulated gene 2 (NDRG2) as a candidate tumour suppressor gene on chromosome 14q11. Here we demonstrate that NDRG2 is a novel PTEN-interacting protein and recruits protein phosphatase 2A to facilitate the dephosphorylation of PTEN at the Ser380, Thr382, and Thr383 (STT) cluster within the C terminus. Although the PI3K/AKT signalling pathway is frequently activated in ATLL cells, neither the genetic alterations of the PTEN and PIK3CA genes nor the transcriptional down-regulation of PTEN expression were detected in ATLL. We found that activation of the PI3K/AKT signalling pathway in ATLL is mediated through hyperphosphorylation of PTEN-STT, a well-known mechanism of PTEN inactivation, by down-regulation of NDRG2 expression. Moreover, we found that NDRG2 deficiency in mice results in constitutive PI3K/AKT activation in various organs and high incidence of T-cell lymphoma and other types of cancer. Since down-regulation of NDRG2 expression via promoter methylation has been reported in various types of cancer, activation of the PI3K/AKT pathway by the enhanced PTEN-STT phosphorylation through the inactivation of NDRG2 is crucial for the development of a wide range of cancer cells including ATLL, which harbor no activating genetic alterations in the PI3K/AKT pathway.

